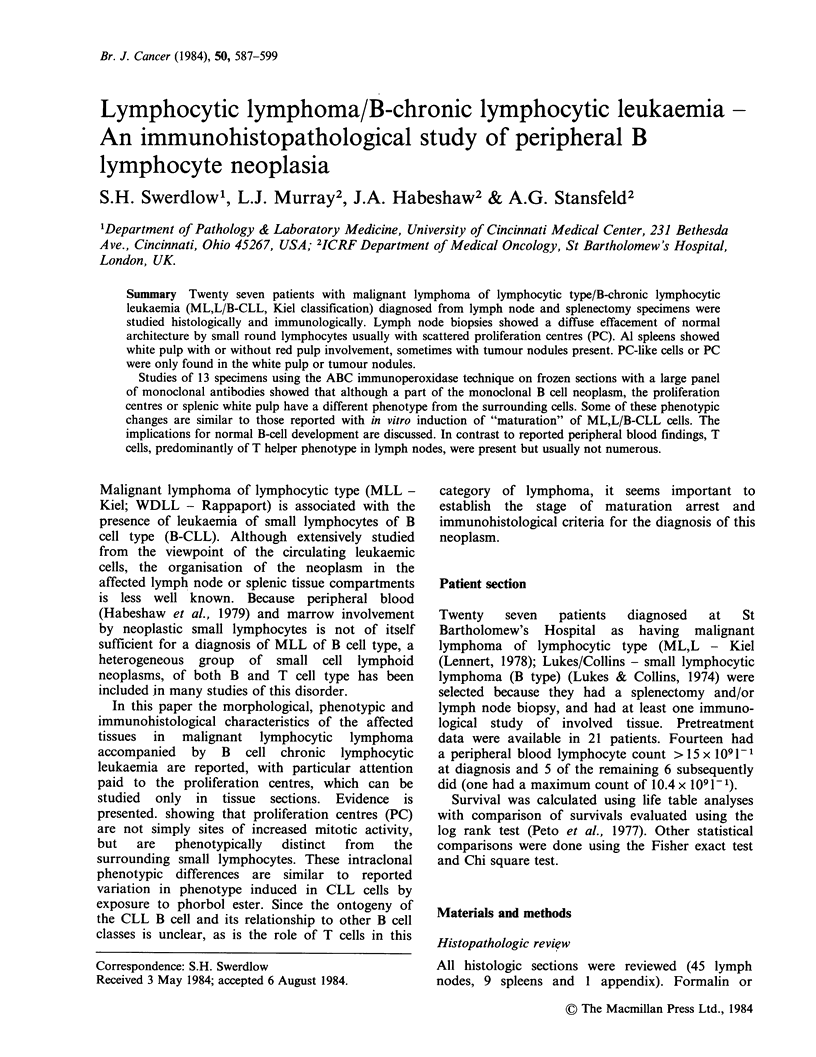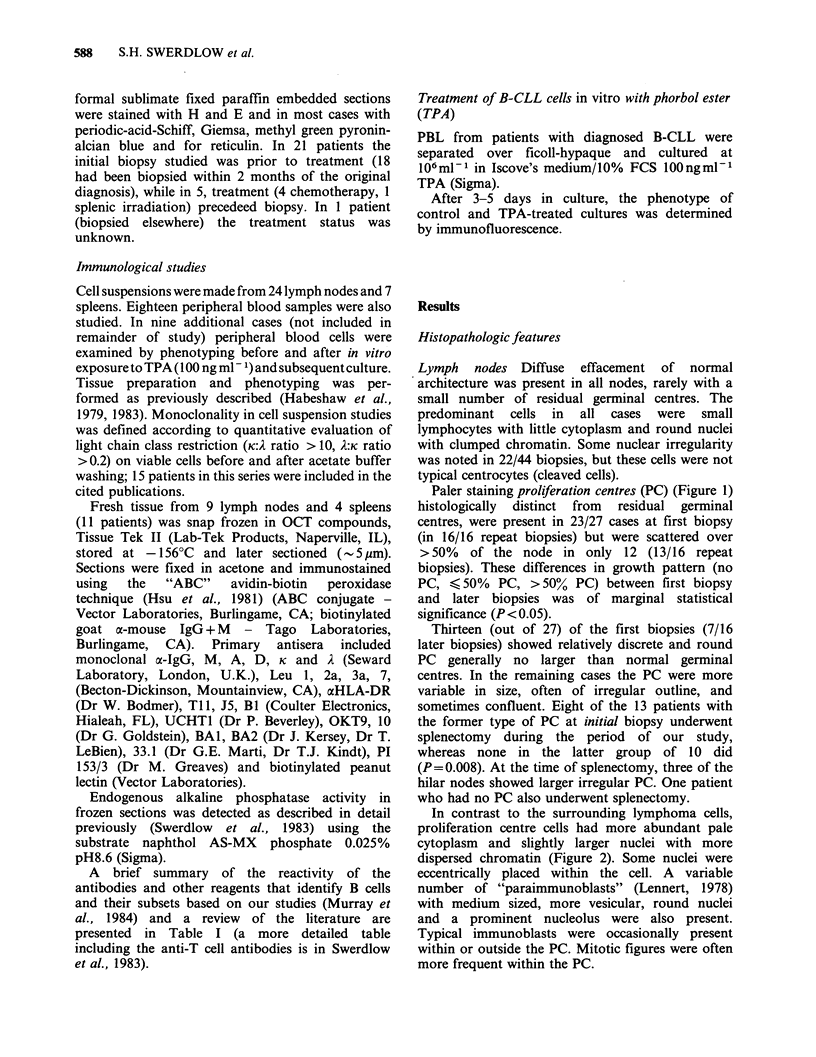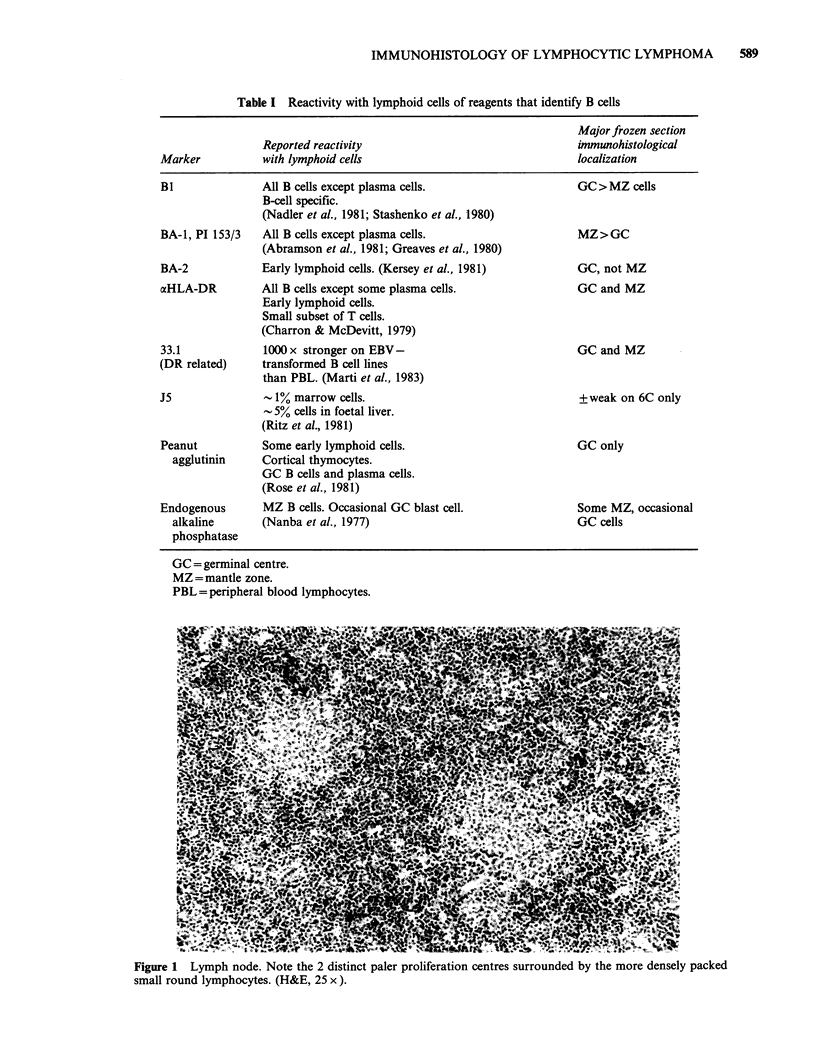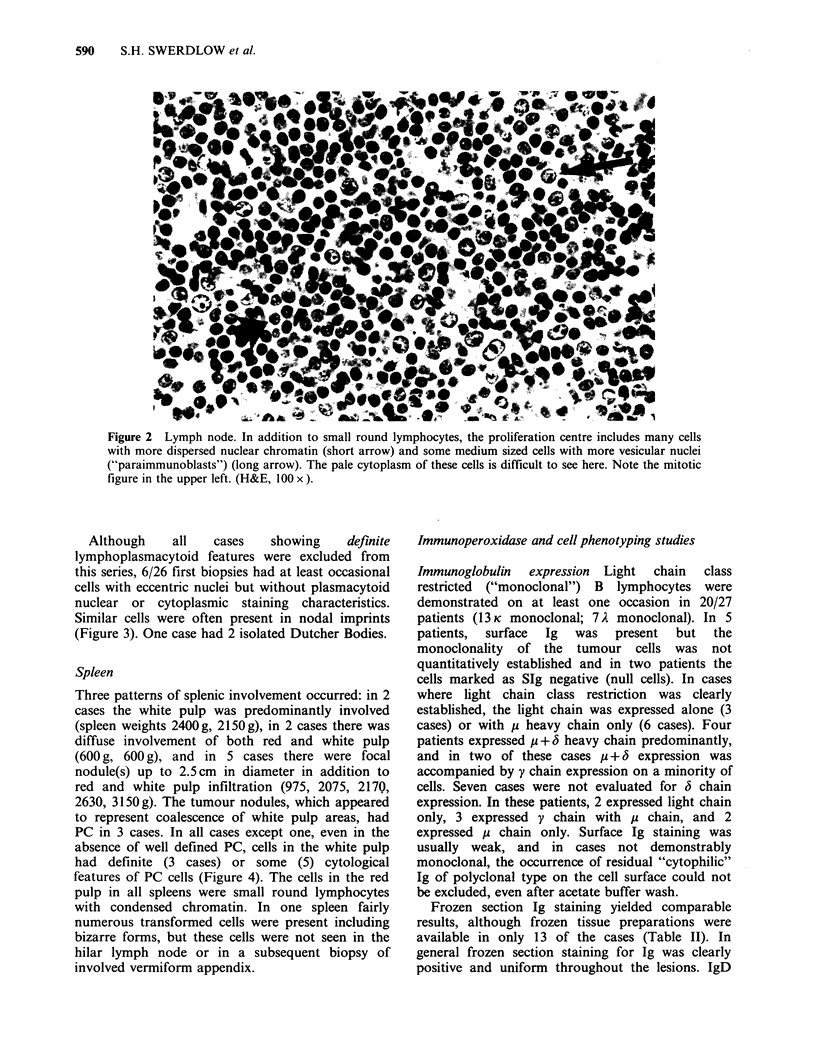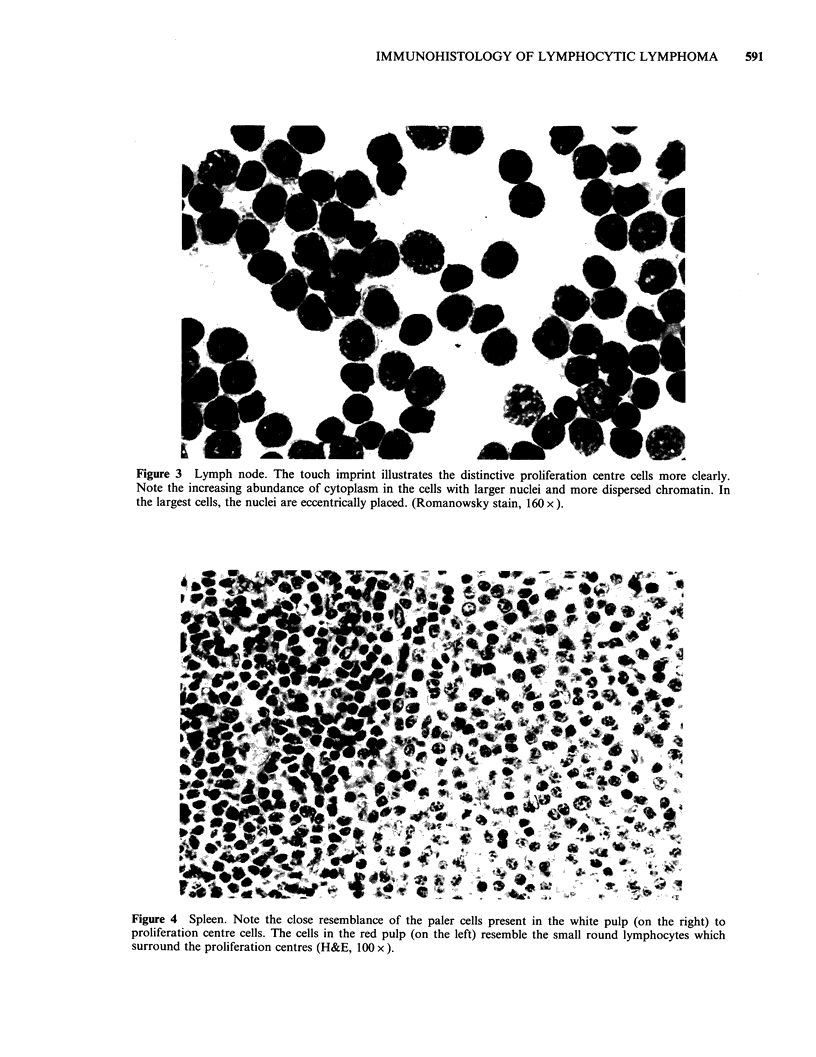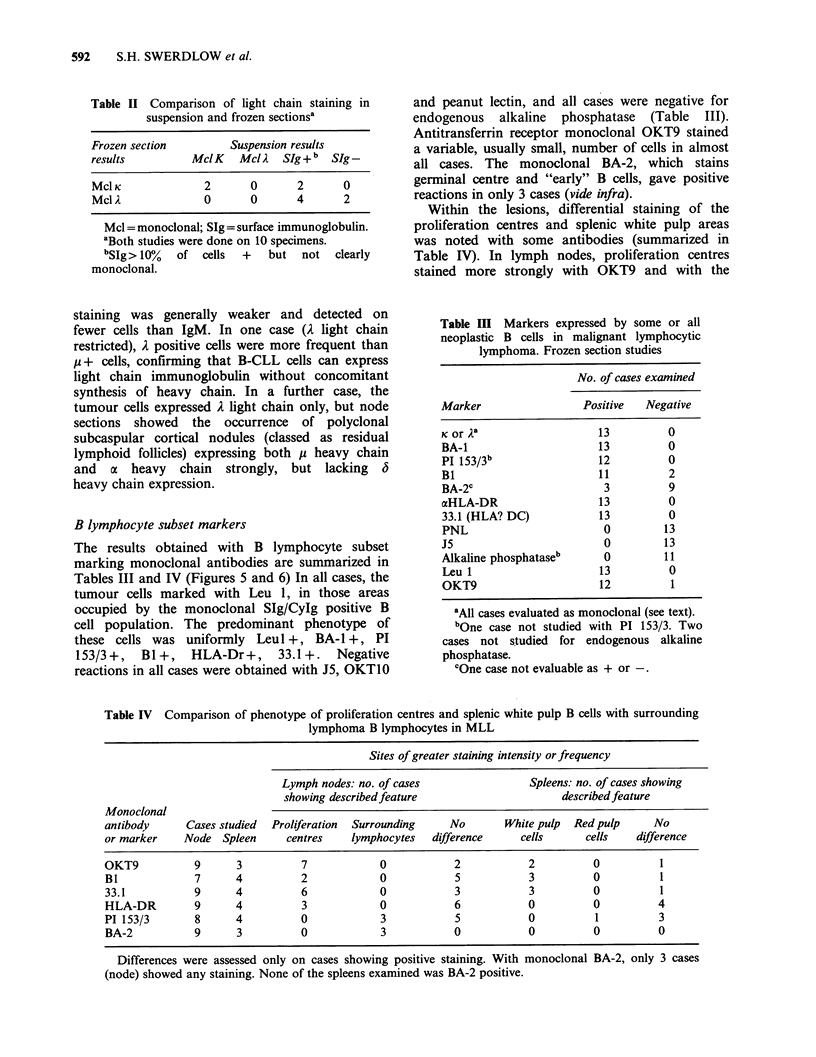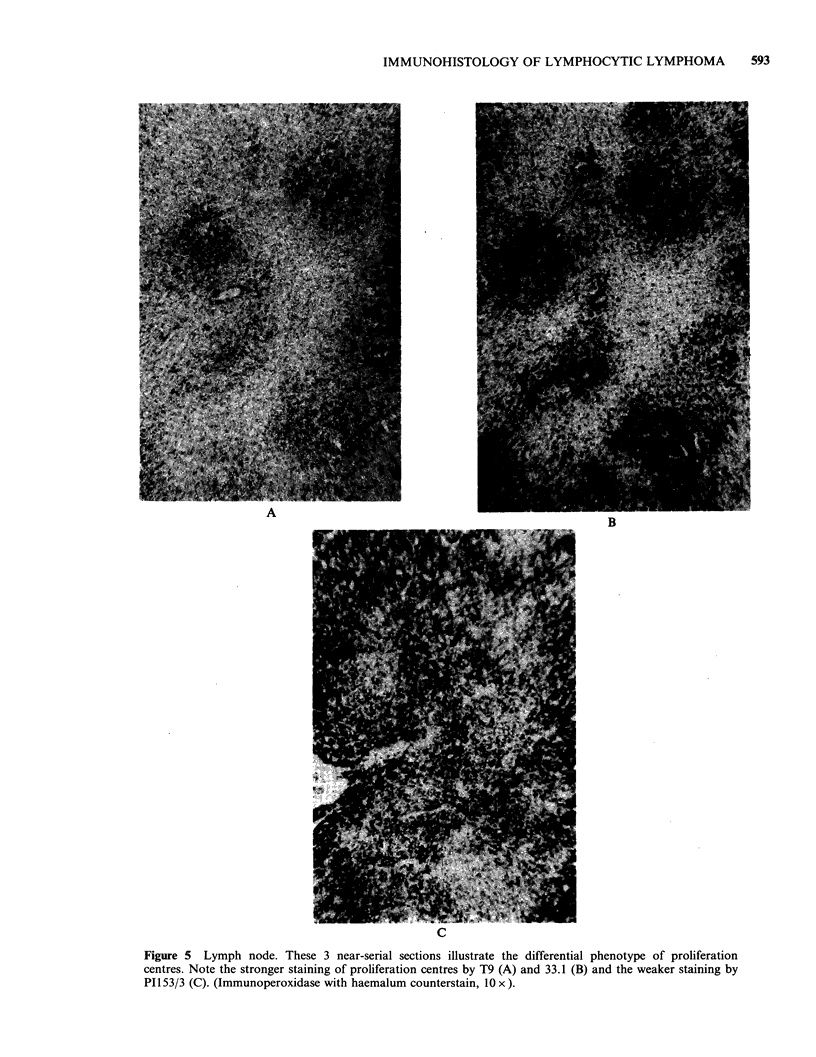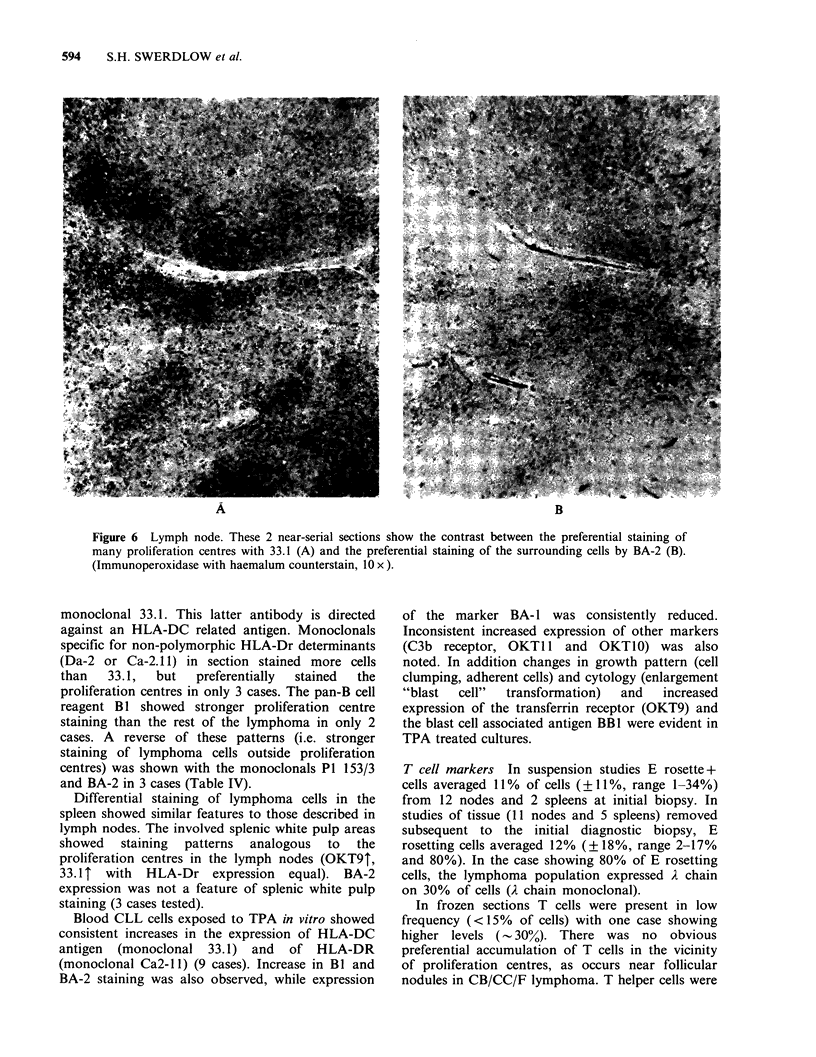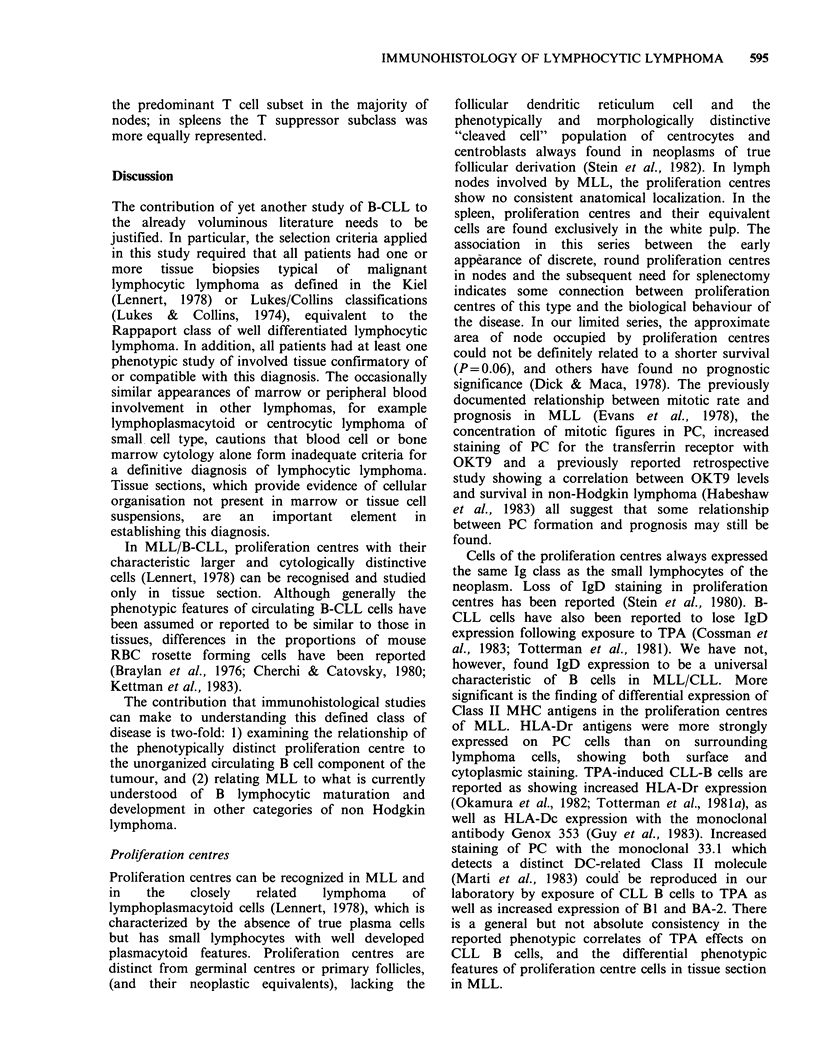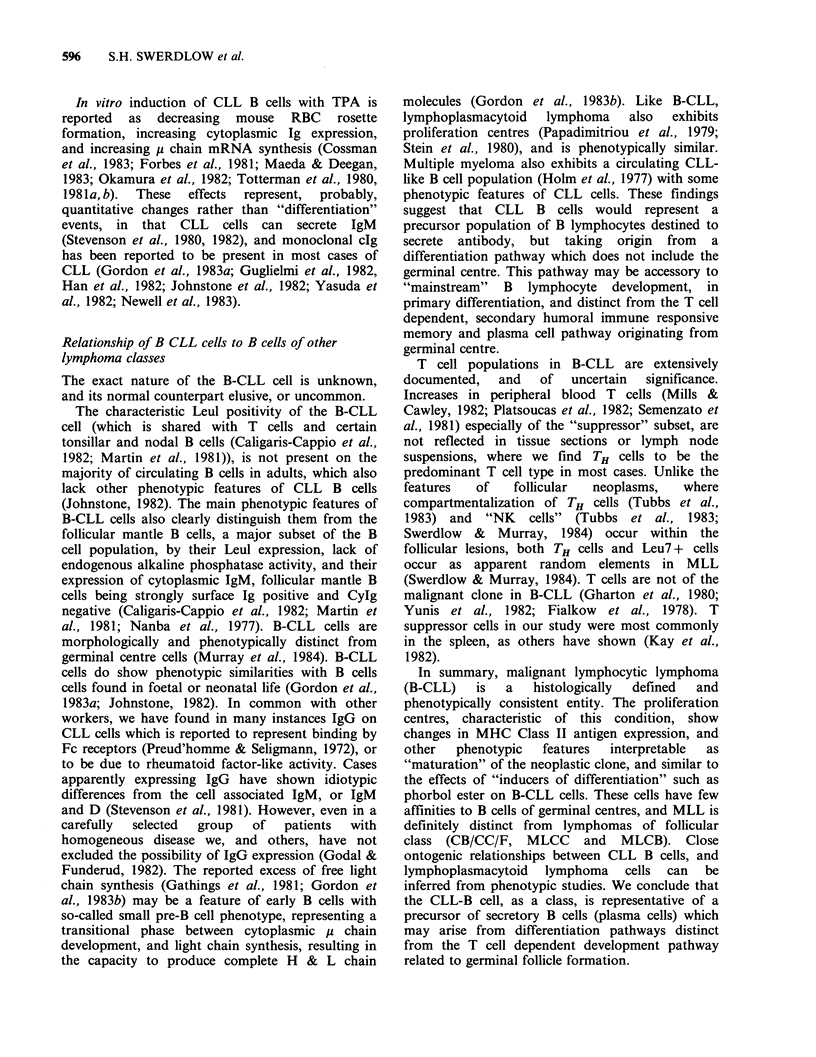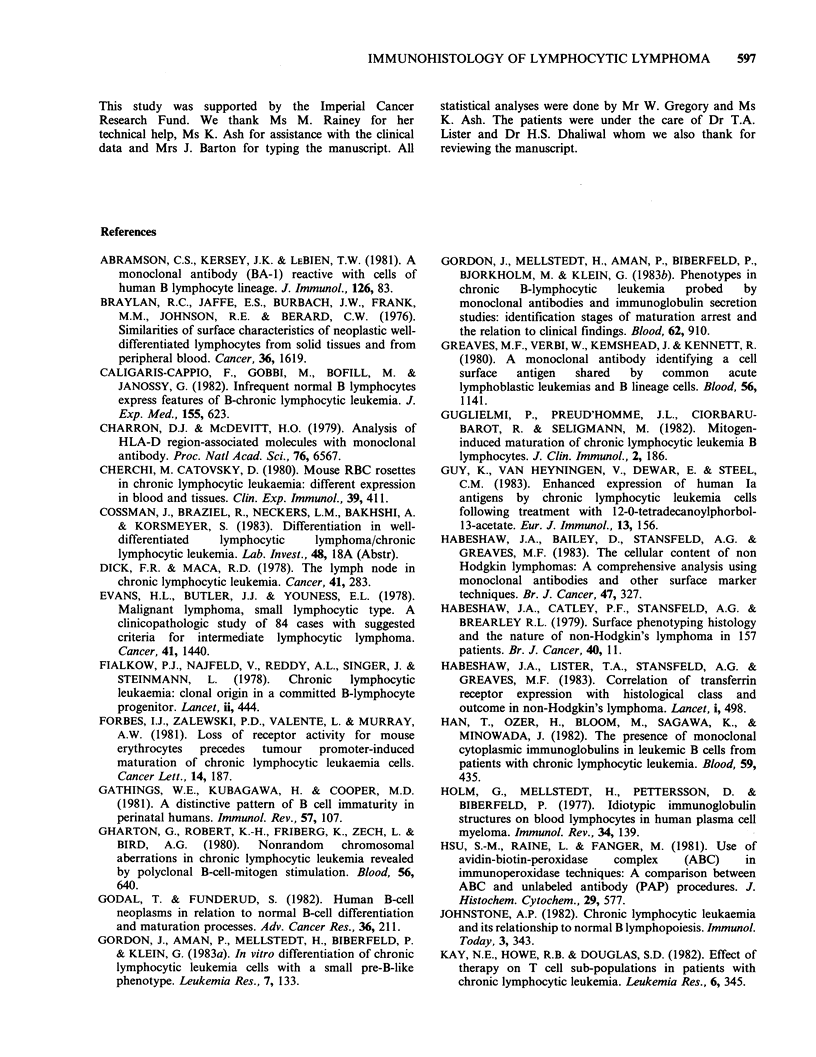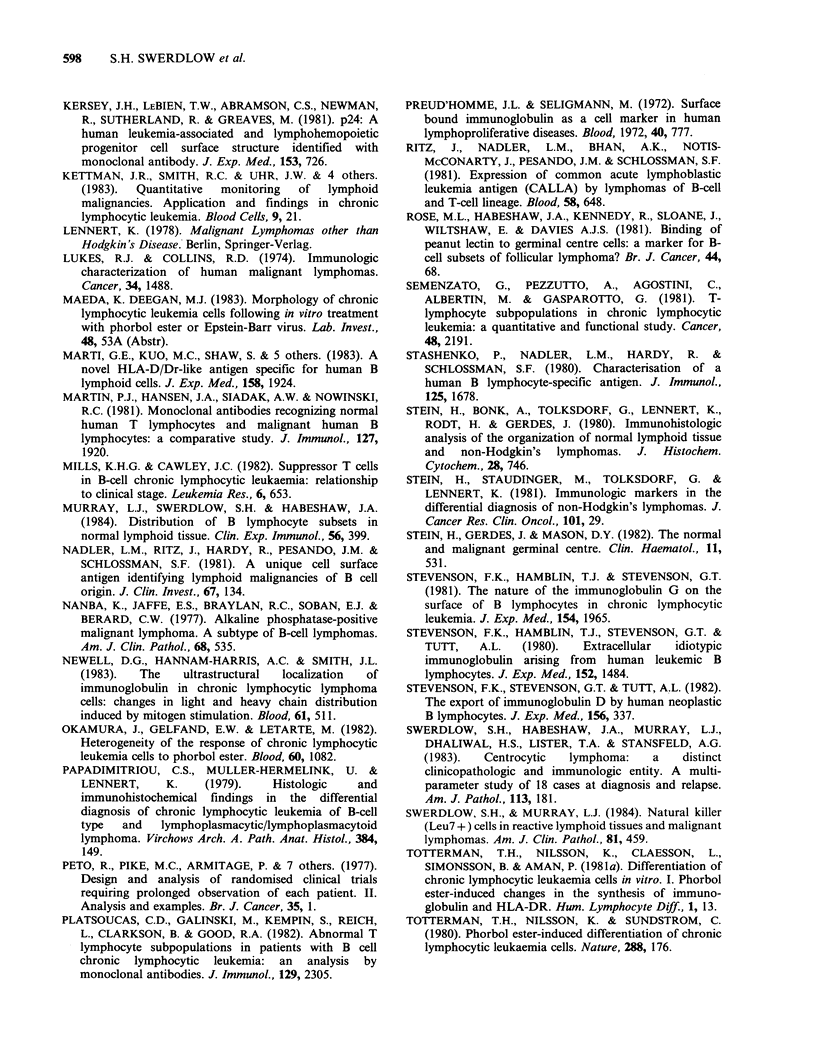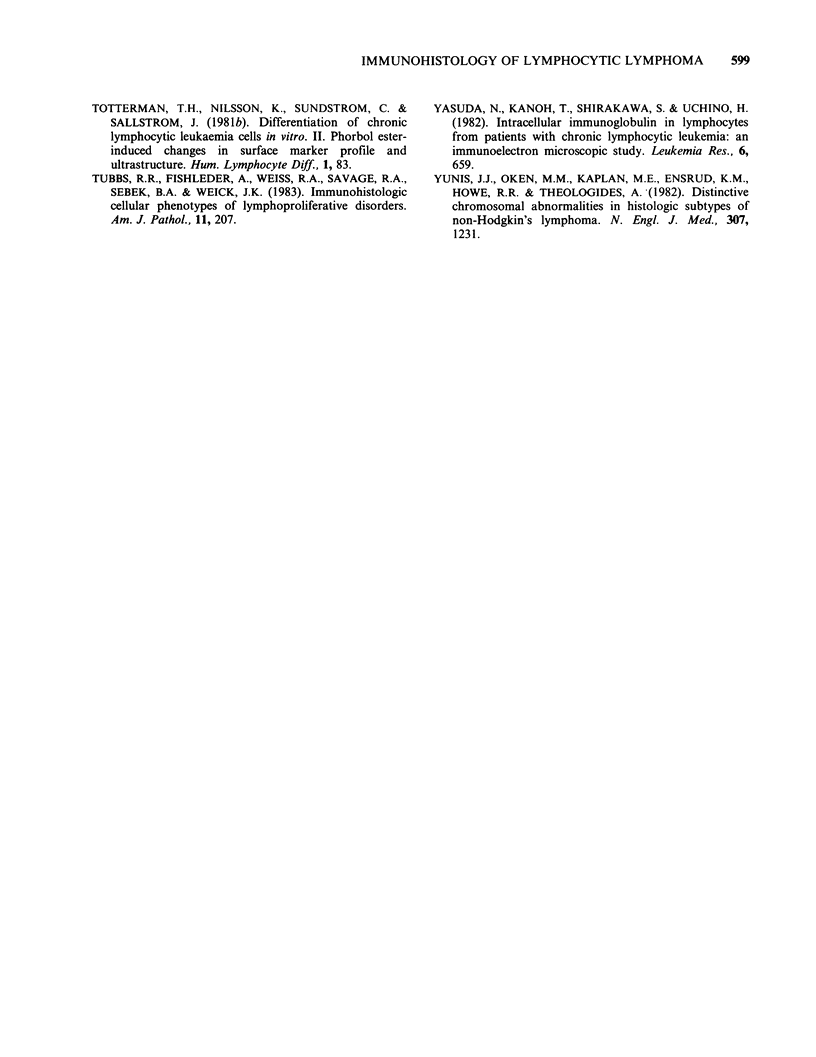# Lymphocytic lymphoma/B-chronic lymphocytic leukaemia--an immunohistopathological study of peripheral B lymphocyte neoplasia.

**DOI:** 10.1038/bjc.1984.225

**Published:** 1984-11

**Authors:** S. H. Swerdlow, L. J. Murray, J. A. Habeshaw, A. G. Stansfeld

## Abstract

**Images:**


Br. J. Cancer (1984), 50, 587-599

### Lymphocytic lymphoma/B-chronic lymphocytic leukaemia – An immunohistopathological study of peripheral B lymphocyte neoplasia

S.H. Swerdlow[1], L.J. Murray[2], J.A. Habeshaw[2] & A.G. Stansfeld[2]

[1]*Department of Pathology & Laboratory Medicine, University of Cincinnati Medical Center, 231 Bethesda Ave., Cincinnati, Ohio 45267, USA;* [2]*ICRF Department of Medical Oncology, St Bartholomew's Hospital, London, UK.*

**Summary** Twenty seven patients with malignant lymphoma of lymphocytic type/B-chronic lymphocytic leukaemia (ML,L/B-CLL, Kiel classification) diagnosed from lymph node and splenectomy specimens were studied histologically and immunologically. Lymph node biopsies showed a diffuse effacement of normal architecture by small round lymphocytes usually with scattered proliferation centres (PC). Al spleens showed white pulp with or without red pulp involvement, sometimes with tumour nodules present. PC-like cells or PC were only found in the white pulp or tumour nodules.

Studies of 13 specimens using the ABC immunoperoxidase technique on frozen sections with a large panel of monoclonal antibodies showed that although a part of the monoclonal B cell neoplasm, the proliferation centres or splenic white pulp have a different phenotype from the surrounding cells. Some of these phenotypic changes are similar to those reported with *in vitro* induction of "maturation" of ML,L/B-CLL cells. The implications for normal B-cell development are discussed. In contrast to reported peripheral blood findings, T cells, predominantly of T helper phenotype in lymph nodes, were present but usually not numerous.

Malignant lymphoma of lymphocytic type (MLL – Kiel; WDLL – Rappaport) is associated with the presence of leukaemia of small lymphocytes of B cell type (B-CLL). Although extensively studied from the viewpoint of the circulating leukaemic cells, the organisation of the neoplasm in the affected lymph node or splenic tissue compartments is less well known. Because peripheral blood (Habeshaw *et al.*, 1979) and marrow involvement by neoplastic small lymphocytes is not of itself sufficient for a diagnosis of MLL of B cell type, a heterogeneous group of small cell lymphoid neoplasms, of both B and T cell type has been included in many studies of this disorder.

In this paper the morphological, phenotypic and immunohistological characteristics of the affected tissues in malignant lymphocytic lymphoma accompanied by B cell chronic lymphocytic leukaemia are reported, with particular attention paid to the proliferation centres, which can be studied only in tissue sections. Evidence is presented. showing that proliferation centres (PC) are not simply sites of increased mitotic activity, but are phenotypically distinct from the surrounding small lymphocytes. These intraclonal phenotypic differences are similar to reported variation in phenotype induced in CLL cells by exposure to phorbol ester. Since the ontogeny of the CLL B cell and its relationship to other B cell classes is unclear, as is the role of T cells in this category of lymphoma, it seems important to establish the stage of maturation arrest and immunohistological criteria for the diagnosis of this neoplasm.

Correspondence: S.H. Swerdlow
Received 3 May 1984; accepted 6 August 1984.

### Patient section

Twenty seven patients diagnosed at St Bartholomew's Hospital as having malignant lymphoma of lymphocytic type (ML,L – Kiel (Lennert, 1978); Lukes/Collins – small lymphocytic lymphoma (B type) (Lukes & Collins, 1974) were selected because they had a splenectomy and/or lymph node biopsy, and had at least one immunological study of involved tissue. Pretreatment data were available in 21 patients. Fourteen had a peripheral blood lymphocyte count $>15\times10^9\,l^{-1}$ at diagnosis and 5 of the remaining 6 subsequently did (one had a maximum count of $10.4\times10^9\,l^{-1}$).

Survival was calculated using life table analyses with comparison of survivals evaluated using the log rank test (Peto *et al.*, 1977). Other statistical comparisons were done using the Fisher exact test and Chi square test.

### Materials and methods

### *Histopathologic review*

All histologic sections were reviewed (45 lymph nodes, 9 spleens and 1 appendix). Formalin or

© The Macmillan Press Ltd., 1984

formal sublimate fixed paraffin embedded sections were stained with H and E and in most cases with periodic-acid-Schiff, Giemsa, methyl green pyronin-alcian blue and for reticulin. In 21 patients the initial biopsy studied was prior to treatment (18 had been biopsied within 2 months of the original diagnosis), while in 5, treatment (4 chemotherapy, 1 splenic irradiation) precedeed biopsy. In 1 patient (biopsied elsewhere) the treatment status was unknown.

*Immunological studies*

Cell suspensions were made from 24 lymph nodes and 7 spleens. Eighteen peripheral blood samples were also studied. In nine additional cases (not included in remainder of study) peripheral blood cells were examined by phenotyping before and after *in vitro* exposure to TPA (100 ng $ml^{-1}$) and subsequent culture. Tissue preparation and phenotyping was performed as previously described (Habeshaw *et al.*, 1979, 1983). Monoclonality in cell suspension studies was defined according to quantitative evaluation of light chain class restriction ($\kappa$:$\lambda$ ratio $>10$, $\lambda$:$\kappa$ ratio $>0.2$) on viable cells before and after acetate buffer washing; 15 patients in this series were included in the cited publications.

Fresh tissue from 9 lymph nodes and 4 spleens (11 patients) was snap frozen in OCT compounds, Tissue Tek II (Lab-Tek Products, Naperville, IL), stored at $-156°C$ and later sectioned ($\sim 5\,\mu m$). Sections were fixed in acetone and immunostained using the "ABC" avidin-biotin peroxidase technique (Hsu *et al.*, 1981) (ABC conjugate – Vector Laboratories, Burlingame, CA; biotinylated goat $\alpha$-mouse IgG+M – Tago Laboratories, Burlingame, CA). Primary antisera included monoclonal $\alpha$-IgG, M, A, D, $\kappa$ and $\lambda$ (Seward Laboratory, London, U.K.), Leu 1, 2a, 3a, 7, (Becton-Dickinson, Mountainview, CA), $\alpha$HLA-DR (Dr W. Bodmer), T11, J5, B1 (Coulter Electronics, Hialeah, FL), UCHT1 (Dr P. Beverley), OKT9, 10 (Dr G. Goldstein), BA1, BA2 (Dr J. Kersey, Dr T. LeBien), 33.1 (Dr G.E. Marti, Dr T.J. Kindt), PI 153/3 (Dr M. Greaves) and biotinylated peanut lectin (Vector Laboratories).

Endogenous alkaline phosphatase activity in frozen sections was detected as described in detail previously (Swerdlow *et al.*, 1983) using the substrate naphthol AS-MX phosphate 0.025% pH8.6 (Sigma).

A brief summary of the reactivity of the antibodies and other reagents that identify B cells and their subsets based on our studies (Murray *et al.*, 1984) and a review of the literature are presented in Table I (a more detailed table including the anti-T cell antibodies is in Swerdlow *et al.*, 1983).

*Treatment of B-CLL cells* in vitro *with phorbol ester (TPA)*

PBL from patients with diagnosed B-CLL were separated over ficoll-hypaque and cultured at $10^6\,ml^{-1}$ in Iscove's medium/10% FCS 100 ng $ml^{-1}$ TPA (Sigma).

After 3–5 days in culture, the phenotype of control and TPA-treated cultures was determined by immunofluorescence.

### Results

*Histopathologic features*

*Lymph nodes* Diffuse effacement of normal architecture was present in all nodes, rarely with a small number of residual germinal centres. The predominant cells in all cases were small lymphocytes with little cytoplasm and round nuclei with clumped chromatin. Some nuclear irregularity was noted in 22/44 biopsies, but these cells were not typical centrocytes (cleaved cells).

Paler staining *proliferation centres* (PC) (Figure 1) histologically distinct from residual germinal centres, were present in 23/27 cases at first biopsy (in 16/16 repeat biopsies) but were scattered over >50% of the node in only 12 (13/16 repeat biopsies). These differences in growth pattern (no PC, ⩽50% PC, >50% PC) between first biopsy and later biopsies was of marginal statistical significance ($P<0.05$).

Thirteen (out of 27) of the first biopsies (7/16 later biopsies) showed relatively discrete and round PC generally no larger than normal germinal centres. In the remaining cases the PC were more variable in size, often of irregular outline, and sometimes confluent. Eight of the 13 patients with the former type of PC at *initial* biopsy underwent splenectomy during the period of our study, whereas none in the latter group of 10 did ($P=0.008$). At the time of splenectomy, three of the hilar nodes showed larger irregular PC. One patient who had no PC also underwent splenectomy.

In contrast to the surrounding lymphoma cells, proliferation centre cells had more abundant pale cytoplasm and slightly larger nuclei with more dispersed chromatin (Figure 2). Some nuclei were eccentrically placed within the cell. A variable number of "paraimmunoblasts" (Lennert, 1978) with medium sized, more vesicular, round nuclei and a prominent nucleolus were also present. Typical immunoblasts were occasionally present within or outside the PC. Mitotic figures were often more frequent within the PC.

**Table I** Reactivity with lymphoid cells of reagents that identify B cells

| *Marker* | *Reported reactivity with lymphoid cells* | *Major frozen section immunohistological localization* |
|---|---|---|
| B1 | All B cells except plasma cells. B-cell specific. (Nadler *et al.*, 1981; Stashenko *et al.*, 1980) | GC>MZ cells |
| BA-1, PI 153/3 | All B cells except plasma cells. (Abramson *et al.*, 1981; Greaves *et al.*, 1980) | MZ>GC |
| BA-2 | Early lymphoid cells. (Kersey *et al.*, 1981) | GC, not MZ |
| αHLA-DR | All B cells except some plasma cells. Early lymphoid cells. Small subset of T cells. (Charron & McDevitt, 1979) | GC and MZ |
| 33.1 (DR related) | 1000× stronger on EBV-transformed B cell lines than PBL. (Marti *et al.*, 1983) | GC and MZ |
| J5 | ~1% marrow cells. ~5% cells in foetal liver. (Ritz *et al.*, 1981) | ±weak on 6C only |
| Peanut agglutinin | Some early lymphoid cells. Cortical thymocytes. GC B cells and plasma cells. (Rose *et al.*, 1981) | GC only |
| Endogenous alkaline phosphatase | MZ B cells. Occasional GC blast cell. (Nanba *et al.*, 1977) | Some MZ, occasional GC cells |

GC = germinal centre.
MZ = mantle zone.
PBL = peripheral blood lymphocytes.

**Figure 1** Lymph node. Note the 2 distinct paler proliferation centres surrounded by the more densely packed small round lymphocytes. (H&E, 25×).

**Figure 2** Lymph node. In addition to small round lymphocytes, the proliferation centre includes many cells with more dispersed nuclear chromatin (short arrow) and some medium sized cells with more vesicular nuclei ("paraimmunoblasts") (long arrow). The pale cytoplasm of these cells is difficult to see here. Note the mitotic figure in the upper left. (H&E, 100 ×).

Although all cases showing *definite* lymphoplasmacytoid features were excluded from this series, 6/26 first biopsies had at least occasional cells with eccentric nuclei but without plasmacytoid nuclear or cytoplasmic staining characteristics. Similar cells were often present in nodal imprints (Figure 3). One case had 2 isolated Dutcher Bodies.

*Spleen*

Three patterns of splenic involvement occurred: in 2 cases the white pulp was predominantly involved (spleen weights 2400 g, 2150 g), in 2 cases there was diffuse involvement of both red and white pulp (600 g, 600 g), and in 5 cases there were focal nodule(s) up to 2.5 cm in diameter in addition to red and white pulp infiltration (975, 2075, 2170, 2630, 3150 g). The tumour nodules, which appeared to represent coalescence of white pulp areas, had PC in 3 cases. In all cases except one, even in the absence of well defined PC, cells in the white pulp had definite (3 cases) or some (5) cytological features of PC cells (Figure 4). The cells in the red pulp in all spleens were small round lymphocytes with condensed chromatin. In one spleen fairly numerous transformed cells were present including bizarre forms, but these cells were not seen in the hilar lymph node or in a subsequent biopsy of involved vermiform appendix.

*Immunoperoxidase and cell phenotyping studies*

*Immunoglobulin expression* Light chain class restricted ("monoclonal") B lymphocytes were demonstrated on at least one occasion in 20/27 patients (13 $\kappa$ monoclonal; 7 $\lambda$ monoclonal). In 5 patients, surface Ig was present but the monoclonality of the tumour cells was not quantitatively established and in two patients the cells marked as SIg negative (null cells). In cases where light chain class restriction was clearly established, the light chain was expressed alone (3 cases) or with $\mu$ heavy chain only (6 cases). Four patients expressed $\mu+\delta$ heavy chain predominantly, and in two of these cases $\mu+\delta$ expression was accompanied by $\gamma$ chain expression on a minority of cells. Seven cases were not evaluated for $\delta$ chain expression. In these patients, 2 expressed light chain only, 3 expressed $\gamma$ chain with $\mu$ chain, and 2 expressed $\mu$ chain only. Surface Ig staining was usually weak, and in cases not demonstrably monoclonal, the occurrence of residual "cytophilic" Ig of polyclonal type on the cell surface could not be excluded, even after acetate buffer wash.

Frozen section Ig staining yielded comparable results, although frozen tissue preparations were available in only 13 of the cases (Table II). In general frozen section staining for Ig was clearly positive and uniform throughout the lesions. IgD

**Figure 3** Lymph node. The touch imprint illustrates the distinctive proliferation centre cells more clearly. Note the increasing abundance of cytoplasm in the cells with larger nuclei and more dispersed chromatin. In the largest cells, the nuclei are eccentrically placed. (Romanowsky stain, 160 × ).

**Figure 4** Spleen. Note the close resemblance of the paler cells present in the white pulp (on the right) to proliferation centre cells. The cells in the red pulp (on the left) resemble the small round lymphocytes which surround the proliferation centres (H&E, 100 × ).

**Table II** Comparison of light chain staining in suspension and frozen sections[a]

| *Frozen section results* | *Suspension results* *Mcl K* | *Mcl λ* | *SIg+*[b] | *SIg−* |
|---|---|---|---|---|
| Mcl κ | 2 | 0 | 2 | 0 |
| Mcl λ | 0 | 0 | 4 | 2 |

Mcl = monoclonal; SIg = surface immunoglobulin.
[a]Both studies were done on 10 specimens.
[b]SIg > 10% of cells + but not clearly monoclonal.

staining was generally weaker and detected on fewer cells than IgM. In one case (λ light chain restricted), λ positive cells were more frequent than μ+ cells, confirming that B-CLL cells can express light chain immunoglobulin without concomitant synthesis of heavy chain. In a further case, the tumour cells expressed λ light chain only, but node sections showed the occurrence of polyclonal subcaspular cortical nodules (classed as residual lymphoid follicles) expressing both μ heavy chain and α heavy chain strongly, but lacking δ heavy chain expression.

### *B lymphocyte subset markers*

The results obtained with B lymphocyte subset marking monoclonal antibodies are summarized in Tables III and IV (Figures 5 and 6) In all cases, the tumour cells marked with Leu 1, in those areas occupied by the monoclonal SIg/CyIg positive B cell population. The predominant phenotype of these cells was uniformly Leu1+, BA-1+, PI 153/3+, B1+, HLA-Dr+, 33.1+. Negative reactions in all cases were obtained with J5, OKT10 and peanut lectin, and all cases were negative for endogenous alkaline phosphatase (Table III). Antitransferrin receptor monoclonal OKT9 stained a variable, usually small, number of cells in almost all cases. The monoclonal BA-2, which stains germinal centre and "early" B cells, gave positive reactions in only 3 cases (*vide infra*).

Within the lesions, differential staining of the proliferation centres and splenic white pulp areas was noted with some antibodies (summarized in Table IV). In lymph nodes, proliferation centres stained more strongly with OKT9 and with the

**Table III** Markers expressed by some or all neoplastic B cells in malignant lymphocytic lymphoma. Frozen section studies

| *Marker* | *No. of cases examined* *Positive* | *Negative* |
|---|---|---|
| κ or λ[a] | 13 | 0 |
| BA-1 | 13 | 0 |
| PI 153/3[b] | 12 | 0 |
| B1 | 11 | 2 |
| BA-2[c] | 3 | 9 |
| αHLA-DR | 13 | 0 |
| 33.1 (HLA? DC) | 13 | 0 |
| PNL | 0 | 13 |
| J5 | 0 | 13 |
| Alkaline phosphatase[b] | 0 | 11 |
| Leu 1 | 13 | 0 |
| OKT9 | 12 | 1 |

[a]All cases evaluated as monoclonal (see text).
[b]One case not studied with PI 153/3. Two cases not studied for endogenous alkaline phosphatase.
[c]One case not evaluable as + or −.

**Table IV** Comparison of phenotype of proliferation centres and splenic white pulp B cells with surrounding lymphoma B lymphocytes in MLL

| | | | *Sites of greater staining intensity or frequency* | | | | | |
|---|---|---|---|---|---|---|---|---|
| | | | *Lymph nodes: no. of cases showing described feature* | | | *Spleens: no. of cases showing described feature* | | |
| *Monoclonal antibody or marker* | *Cases studied Node* | *Spleen* | *Proliferation centres* | *Surrounding lymphocytes* | *No difference* | *White pulp cells* | *Red pulp cells* | *No difference* |
| OKT9 | 9 | 3 | 7 | 0 | 2 | 2 | 0 | 1 |
| B1 | 7 | 4 | 2 | 0 | 5 | 3 | 0 | 1 |
| 33.1 | 9 | 4 | 6 | 0 | 3 | 3 | 0 | 1 |
| HLA-DR | 9 | 4 | 3 | 0 | 6 | 0 | 0 | 4 |
| PI 153/3 | 8 | 4 | 0 | 3 | 5 | 0 | 1 | 3 |
| BA-2 | 9 | 3 | 0 | 3 | 0 | 0 | 0 | 0 |

Differences were assessed only on cases showing positive staining. With monoclonal BA-2, only 3 cases (node) showed any staining. None of the spleens examined was BA-2 positive.

**Figure 5** Lymph node. These 3 near-serial sections illustrate the differential phenotype of proliferation centres. Note the stronger staining of proliferation centres by T9 (A) and 33.1 (B) and the weaker staining by PI153/3 (C). (Immunoperoxidase with haemalum counterstain, 10 ×).

A B

**Figure 6** Lymph node. These 2 near-serial sections show the contrast between the preferential staining of many proliferation centres with 33.1 (A) and the preferential staining of the surrounding cells by BA-2 (B). (Immunoperoxidase with haemalum counterstain, 10 × ).

monoclonal 33.1. This latter antibody is directed against an HLA-DC related antigen. Monoclonals specific for non-polymorphic HLA-Dr determinants (Da-2 or Ca-2.11) in section stained more cells than 33.1, but preferentially stained the proliferation centres in only 3 cases. The pan-B cell reagent B1 showed stronger proliferation centre staining than the rest of the lymphoma in only 2 cases. A reverse of these patterns (i.e. stronger staining of lymphoma cells outside proliferation centres) was shown with the monoclonals P1 153/3 and BA-2 in 3 cases (Table IV).

Differential staining of lymphoma cells in the spleen showed similar features to those described in lymph nodes. The involved splenic white pulp areas showed staining patterns analogous to the proliferation centres in the lymph nodes (OKT9↑, 33.1↑ with HLA-Dr expression equal). BA-2 expression was not a feature of splenic white pulp staining (3 cases tested).

Blood CLL cells exposed to TPA *in vitro* showed consistent increases in the expression of HLA-DC antigen (monoclonal 33.1) and of HLA-DR (monoclonal Ca2-11) (9 cases). Increase in B1 and BA-2 staining was also observed, while expression of the marker BA-1 was consistently reduced. Inconsistent increased expression of other markers (C3b receptor, OKT11 and OKT10) was also noted. In addition changes in growth pattern (cell clumping, adherent cells) and cytology (enlargement "blast cell" transformation) and increased expression of the transferrin receptor (OKT9) and the blast cell associated antigen BB1 were evident in TPA treated cultures.

*T cell markers* In suspension studies E rosette+ cells averaged 11% of cells (±11%, range 1–34%) from 12 nodes and 2 spleens at initial biopsy. In studies of tissue (11 nodes and 5 spleens) removed subsequent to the initial diagnostic biopsy, E rosetting cells averaged 12% (±18%, range 2–17% and 80%). In the case showing 80% of E rosetting cells, the lymphoma population expressed λ chain on 30% of cells (λ chain monoclonal).

In frozen sections T cells were present in low frequency (<15% of cells) with one case showing higher levels (~30%). There was no obvious preferential accumulation of T cells in the vicinity of proliferation centres, as occurs near follicular nodules in CB/CC/F lymphoma. T helper cells were the predominant T cell subset in the majority of nodes; in spleens the T suppressor subclass was more equally represented.

### Discussion

The contribution of yet another study of B-CLL to the already voluminous literature needs to be justified. In particular, the selection criteria applied in this study required that all patients had one or more tissue biopsies typical of malignant lymphocytic lymphoma as defined in the Kiel (Lennert, 1978) or Lukes/Collins classifications (Lukes & Collins, 1974), equivalent to the Rappaport class of well differentiated lymphocytic lymphoma. In addition, all patients had at least one phenotypic study of involved tissue confirmatory of or compatible with this diagnosis. The occasionally similar appearances of marrow or peripheral blood involvement in other lymphomas, for example lymphoplasmacytoid or centrocytic lymphoma of small cell type, cautions that blood cell or bone marrow cytology alone form inadequate criteria for a definitive diagnosis of lymphocytic lymphoma. Tissue sections, which provide evidence of cellular organisation not present in marrow or tissue cell suspensions, are an important element in establishing this diagnosis.

In MLL/B-CLL, proliferation centres with their characteristic larger and cytologically distinctive cells (Lennert, 1978) can be recognised and studied only in tissue section. Although generally the phenotypic features of circulating B-CLL cells have been assumed or reported to be similar to those in tissues, differences in the proportions of mouse RBC rosette forming cells have been reported (Braylan *et al.*, 1976; Cherchi & Catovsky, 1980; Kettman *et al.*, 1983).

The contribution that immunohistological studies can make to understanding this defined class of disease is two-fold: 1) examining the relationship of the phenotypically distinct proliferation centre to the unorganized circulating B cell component of the tumour, and (2) relating MLL to what is currently understood of B lymphocytic maturation and development in other categories of non Hodgkin lymphoma.

### *Proliferation centres*

Proliferation centres can be recognized in MLL and in the closely related lymphoma of lymphoplasmacytoid cells (Lennert, 1978), which is characterized by the absence of true plasma cells but has small lymphocytes with well developed plasmacytoid features. Proliferation centres are distinct from germinal centres or primary follicles, (and their neoplastic equivalents), lacking the follicular dendritic reticulum cell and the phenotypically and morphologically distinctive "cleaved cell" population of centrocytes and centroblasts always found in neoplasms of true follicular derivation (Stein *et al.*, 1982). In lymph nodes involved by MLL, the proliferation centres show no consistent anatomical localization. In the spleen, proliferation centres and their equivalent cells are found exclusively in the white pulp. The association in this series between the early appearance of discrete, round proliferation centres in nodes and the subsequent need for splenectomy indicates some connection between proliferation centres of this type and the biological behaviour of the disease. In our limited series, the approximate area of node occupied by proliferation centres could not be definitely related to a shorter survival ($P = 0.06$), and others have found no prognostic significance (Dick & Maca, 1978). The previously documented relationship between mitotic rate and prognosis in MLL (Evans *et al.*, 1978), the concentration of mitotic figures in PC, increased staining of PC for the transferrin receptor with OKT9 and a previously reported retrospective study showing a correlation between OKT9 levels and survival in non-Hodgkin lymphoma (Habeshaw *et al.*, 1983) all suggest that some relationship between PC formation and prognosis may still be found.

Cells of the proliferation centres always expressed the same Ig class as the small lymphocytes of the neoplasm. Loss of IgD staining in proliferation centres has been reported (Stein *et al.*, 1980). B-CLL cells have also been reported to lose IgD expression following exposure to TPA (Cossman *et al.*, 1983; Totterman *et al.*, 1981). We have not, however, found IgD expression to be a universal characteristic of B cells in MLL/CLL. More significant is the finding of differential expression of Class II MHC antigens in the proliferation centres of MLL. HLA-Dr antigens were more strongly expressed on PC cells than on surrounding lymphoma cells, showing both surface and cytoplasmic staining. TPA-induced CLL-B cells are reported as showing increased HLA-Dr expression (Okamura *et al.*, 1982; Totterman *et al.*, 1981*a*), as well as HLA-Dc expression with the monoclonal antibody Genox 353 (Guy *et al.*, 1983). Increased staining of PC with the monoclonal 33.1 which detects a distinct DC-related Class II molecule (Marti *et al.*, 1983) could be reproduced in our laboratory by exposure of CLL B cells to TPA as well as increased expression of B1 and BA-2. There is a general but not absolute consistency in the reported phenotypic correlates of TPA effects on CLL B cells, and the differential phenotypic features of proliferation centre cells in tissue section in MLL.

*In vitro* induction of CLL B cells with TPA is reported as decreasing mouse RBC rosette formation, increasing cytoplasmic Ig expression, and increasing $\mu$ chain mRNA synthesis (Cossman *et al.*, 1983; Forbes *et al.*, 1981; Maeda & Deegan, 1983; Okamura *et al.*, 1982; Totterman *et al.*, 1980, 1981*a,b*). These effects represent, probably, quantitative changes rather than "differentiation" events, in that CLL cells can secrete IgM (Stevenson *et al.*, 1980, 1982), and monoclonal cIg has been reported to be present in most cases of CLL (Gordon *et al.*, 1983*a*; Guglielmi *et al.*, 1982, Han *et al.*, 1982; Johnstone *et al.*, 1982; Yasuda *et al.*, 1982; Newell *et al.*, 1983).

*Relationship of B CLL cells to B cells of other lymphoma classes*

The exact nature of the B-CLL cell is unknown, and its normal counterpart elusive, or uncommon.

The characteristic Leul positivity of the B-CLL cell (which is shared with T cells and certain tonsillar and nodal B cells (Caligaris-Cappio *et al.*, 1982; Martin *et al.*, 1981)), is not present on the majority of circulating B cells in adults, which also lack other phenotypic features of CLL B cells (Johnstone, 1982). The main phenotypic features of B-CLL cells also clearly distinguish them from the follicular mantle B cells, a major subset of the B cell population, by their Leul expression, lack of endogenous alkaline phosphatase activity, and their expression of cytoplasmic IgM, follicular mantle B cells being strongly surface Ig positive and CyIg negative (Caligaris-Cappio *et al.*, 1982; Martin *et al.*, 1981; Nanba *et al.*, 1977). B-CLL cells are morphologically and phenotypically distinct from germinal centre cells (Murray *et al.*, 1984). B-CLL cells do show phenotypic similarities with B cells cells found in foetal or neonatal life (Gordon *et al.*, 1983*a*; Johnstone, 1982). In common with other workers, we have found in many instances IgG on CLL cells which is reported to represent binding by Fc receptors (Preud'homme & Seligmann, 1972), or to be due to rheumatoid factor-like activity. Cases apparently expressing IgG have shown idiotypic differences from the cell associated IgM, or IgM and D (Stevenson *et al.*, 1981). However, even in a carefully selected group of patients with homogeneous disease we, and others, have not excluded the possibility of IgG expression (Godal & Funderud, 1982). The reported excess of free light chain synthesis (Gathings *et al.*, 1981; Gordon *et al.*, 1983*b*) may be a feature of early B cells with so-called small pre-B cell phenotype, representing a transitional phase between cytoplasmic $\mu$ chain development, and light chain synthesis, resulting in the capacity to produce complete H & L chain molecules (Gordon *et al.*, 1983*b*). Like B-CLL, lymphoplasmacytoid lymphoma also exhibits proliferation centres (Papadimitriou *et al.*, 1979; Stein *et al.*, 1980), and is phenotypically similar. Multiple myeloma also exhibits a circulating CLL-like B cell population (Holm *et al.*, 1977) with some phenotypic features of CLL cells. These findings suggest that CLL B cells would represent a precursor population of B lymphocytes destined to secrete antibody, but taking origin from a differentiation pathway which does not include the germinal centre. This pathway may be accessory to "mainstream" B lymphocyte development, in primary differentiation, and distinct from the T cell dependent, secondary humoral immune responsive memory and plasma cell pathway originating from germinal centre.

T cell populations in B-CLL are extensively documented, and of uncertain significance. Increases in peripheral blood T cells (Mills & Cawley, 1982; Platsoucas *et al.*, 1982; Semenzato *et al.*, 1981) especially of the "suppressor" subset, are not reflected in tissue sections or lymph node suspensions, where we find $T_H$ cells to be the predominant T cell type in most cases. Unlike the features of follicular neoplasms, where compartmentalization of $T_H$ cells (Tubbs *et al.*, 1983) and "NK cells" (Tubbs *et al.*, 1983; Swerdlow & Murray, 1984) occur within the follicular lesions, both $T_H$ cells and Leu7+ cells occur as apparent random elements in MLL (Swerdlow & Murray, 1984). T cells are not of the malignant clone in B-CLL (Gharton *et al.*, 1980; Yunis *et al.*, 1982; Fialkow *et al.*, 1978). T suppressor cells in our study were most commonly in the spleen, as others have shown (Kay *et al.*, 1982).

In summary, malignant lymphocytic lymphoma (B-CLL) is a histologically defined and phenotypically consistent entity. The proliferation centres, characteristic of this condition, show changes in MHC Class II antigen expression, and other phenotypic features interpretable as "maturation" of the neoplastic clone, and similar to the effects of "inducers of differentiation" such as phorbol ester on B-CLL cells. These cells have few affinities to B cells of germinal centres, and MLL is definitely distinct from lymphomas of follicular class (CB/CC/F, MLCC and MLCB). Close ontogenic relationships between CLL B cells, and lymphoplasmacytoid lymphoma cells can be inferred from phenotypic studies. We conclude that the CLL-B cell, as a class, is representative of a precursor of secretory B cells (plasma cells) which may arise from differentiation pathways distinct from the T cell dependent development pathway related to germinal follicle formation.

This study was supported by the Imperial Cancer Research Fund. We thank Ms M. Rainey for her technical help, Ms K. Ash for assistance with the clinical data and Mrs J. Barton for typing the manuscript. All statistical analyses were done by Mr W. Gregory and Ms K. Ash. The patients were under the care of Dr T.A. Lister and Dr H.S. Dhaliwal whom we also thank for reviewing the manuscript.

### References

ABRAMSON, C.S., KERSEY, J.K. & LEBIEN, T.W. (1981). A monoclonal antibody (BA-1) reactive with cells of human B lymphocyte lineage. *J. Immunol.*, **126**, 83.

BRAYLAN, R.C., JAFFE, E.S., BURBACH, J.W., FRANK, M.M., JOHNSON, R.E. & BERARD, C.W. (1976). Similarities of surface characteristics of neoplastic well-differentiated lymphocytes from solid tissues and from peripheral blood. *Cancer*, **36**, 1619.

CALIGARIS-CAPPIO, F., GOBBI, M., BOFILL, M. & JANOSSY, G. (1982). Infrequent normal B lymphocytes express features of B-chronic lymphocytic leukemia. *J. Exp. Med.*, **155**, 623.

CHARRON, D.J. & MCDEVITT, H.O. (1979). Analysis of HLA-D region-associated molecules with monoclonal antibody. *Proc. Natl Acad. Sci.*, **76**, 6567.

CHERCHI, M. CATOVSKY, D. (1980). Mouse RBC rosettes in chronic lymphocytic leukaemia: different expression in blood and tissues. *Clin. Exp. Immunol.*, **39**, 411.

COSSMAN, J., BRAZIEL, R., NECKERS, L.M., BAKHSHI, A. & KORSMEYER, S. (1983). Differentiation in well-differentiated lymphocytic lymphoma/chronic lymphocytic leukemia. *Lab. Invest.*, **48**, 18A (Abstr).

DICK, F.R. & MACA, R.D. (1978). The lymph node in chronic lymphocytic leukemia. *Cancer*, **41**, 283.

EVANS, H.L., BUTLER, J.J. & YOUNESS, E.L. (1978). Malignant lymphoma, small lymphocytic type. A clinicopathologic study of 84 cases with suggested criteria for intermediate lymphocytic lymphoma. *Cancer*, **41**, 1440.

FIALKOW, P.J., NAJFELD, V., REDDY, A.L., SINGER, J. & STEINMANN, L. (1978). Chronic lymphocytic leukaemia: clonal origin in a committed B-lymphocyte progenitor. *Lancet*, **ii**, 444.

FORBES, I.J., ZALEWSKI, P.D., VALENTE, L. & MURRAY, A.W. (1981). Loss of receptor activity for mouse erythrocytes precedes tumour promoter-induced maturation of chronic lymphocytic leukaemia cells. *Cancer Lett.*, **14**, 187.

GATHINGS, W.E., KUBAGAWA, H. & COOPER, M.D. (1981). A distinctive pattern of B cell immaturity in perinatal humans. *Immunol. Rev.*, **57**, 107.

GHARTON, G., ROBERT, K.-H., FRIBERG, K., ZECH, L. & BIRD, A.G. (1980). Nonrandom chromosomal aberrations in chronic lymphocytic leukemia revealed by polyclonal B-cell-mitogen stimulation. *Blood*, **56**, 640.

GODAL, T. & FUNDERUD, S. (1982). Human B-cell neoplasms in relation to normal B-cell differentiation and maturation processes. *Adv. Cancer Res.*, **36**, 211.

GORDON, J., AMAN, P., MELLSTEDT, H., BIBERFELD, P. & KLEIN, G. (1983*a*). *In vitro* differentiation of chronic lymphocytic leukemia cells with a small pre-B-like phenotype. *Leukemia Res.*, **7**, 133.

GORDON, J., MELLSTEDT, H., AMAN, P., BIBERFELD, P., BJORKHOLM, M. & KLEIN, G. (1983*b*). Phenotypes in chronic B-lymphocytic leukemia probed by monoclonal antibodies and immunoglobulin secretion studies: identification stages of maturation arrest and the relation to clinical findings. *Blood*, **62**, 910.

GREAVES, M.F., VERBI, W., KEMSHEAD, J. & KENNETT, R. (1980). A monoclonal antibody identifying a cell surface antigen shared by common acute lymphoblastic leukemias and B lineage cells. *Blood*, **56**, 1141.

GUGLIELMI, P., PREUD'HOMME, J.L., CIORBARU-BAROT, R. & SELIGMANN, M. (1982). Mitogen-induced maturation of chronic lymphocytic leukemia B lymphocytes. *J. Clin. Immunol.*, **2**, 186.

GUY, K., VAN HEYNINGEN, V., DEWAR, E. & STEEL, C.M. (1983). Enhanced expression of human Ia antigens by chronic lymphocytic leukemia cells following treatment with 12-0-tetradecanoylphorbol-13-acetate. *Eur. J. Immunol.*, **13**, 156.

HABESHAW, J.A., BAILEY, D., STANSFELD, A.G. & GREAVES, M.F. (1983). The cellular content of non Hodgkin lymphomas: A comprehensive analysis using monoclonal antibodies and other surface marker techniques. *Br. J. Cancer*, **47**, 327.

HABESHAW, J.A., CATLEY, P.F., STANSFELD, A.G. & BREARLEY R.L. (1979). Surface phenotyping histology and the nature of non-Hodgkin's lymphoma in 157 patients. *Br. J. Cancer*, **40**, 11.

HABESHAW, J.A., LISTER, T.A., STANSFELD, A.G. & GREAVES, M.F. (1983). Correlation of transferrin receptor expression with histological class and outcome in non-Hodgkin's lymphoma. *Lancet*, **i**, 498.

HAN, T., OZER, H., BLOOM, M., SAGAWA, K., & MINOWADA, J. (1982). The presence of monoclonal cytoplasmic immunoglobulins in leukemic B cells from patients with chronic lymphocytic leukemia. *Blood*, **59**, 435.

HOLM, G., MELLSTEDT, H., PETTERSSON, D. & BIBERFELD, P. (1977). Idiotypic immunoglobulin structures on blood lymphocytes in human plasma cell myeloma. *Immunol. Rev.*, **34**, 139.

HSU, S.-M., RAINE, L. & FANGER, M. (1981). Use of avidin-biotin-peroxidase complex (ABC) in immunoperoxidase techniques: A comparison between ABC and unlabeled antibody (PAP) procedures. *J. Histochem. Cytochem.*, **29**, 577.

JOHNSTONE, A.P. (1982). Chronic lymphocytic leukaemia and its relationship to normal B lymphopoiesis. *Immunol. Today*, **3**, 343.

KAY, N.E., HOWE, R.B. & DOUGLAS, S.D. (1982). Effect of therapy on T cell sub-populations in patients with chronic lymphocytic leukemia. *Leukemia Res.*, **6**, 345.

KERSEY, J.H., LEBIEN, T.W., ABRAMSON, C.S., NEWMAN, R., SUTHERLAND, R. & GREAVES, M. (1981). p24: A human leukemia-associated and lymphohemopoietic progenitor cell surface structure identified with monoclonal antibody. *J. Exp. Med.*, **153**, 726.

KETTMAN, J.R., SMITH, R.C. & UHR, J.W. & 4 others. (1983). Quantitative monitoring of lymphoid malignancies. Application and findings in chronic lymphocytic leukemia. *Blood Cells*, **9**, 21.

LENNERT, K. (1978). *Malignant Lymphomas other than Hodgkin's Disease.* Berlin, Springer-Verlag.

LUKES, R.J. & COLLINS, R.D. (1974). Immunologic characterization of human malignant lymphomas. *Cancer*, **34**, 1488.

MAEDA, K. DEEGAN, M.J. (1983). Morphology of chronic lymphocytic leukemia cells following *in vitro* treatment with phorbol ester or Epstein-Barr virus. *Lab. Invest.*, **48**, 53A (Abstr).

MARTI, G.E., KUO, M.C., SHAW, S. & 5 others. (1983). A novel HLA-D/Dr-like antigen specific for human B lymphoid cells. *J. Exp. Med.*, **158**, 1924.

MARTIN, P.J., HANSEN, J.A., SIADAK, A.W. & NOWINSKI, R.C. (1981). Monoclonal antibodies recognizing normal human T lymphocytes and malignant human B lymphocytes: a comparative study. *J. Immunol.*, **127**, 1920.

MILLS, K.H.G. & CAWLEY, J.C. (1982). Suppressor T cells in B-cell chronic lymphocytic leukaemia: relationship to clinical stage. *Leukemia Res.*, **6**, 653.

MURRAY, L.J., SWERDLOW, S.H. & HABESHAW, J.A. (1984). Distribution of B lymphocyte subsets in normal lymphoid tissue. *Clin. Exp. Immunol.*, **56**, 399.

NADLER, L.M., RITZ, J., HARDY, R., PESANDO, J.M. & SCHLOSSMAN, S.F. (1981). A unique cell surface antigen identifying lymphoid malignancies of B cell origin. *J. Clin. Invest.*, **67**, 134.

NANBA, K., JAFFE, E.S., BRAYLAN, R.C., SOBAN, E.J. & BERARD, C.W. (1977). Alkaline phosphatase-positive malignant lymphoma. A subtype of B-cell lymphomas. *Am. J. Clin. Pathol.*, **68**, 535.

NEWELL, D.G., HANNAM-HARRIS, A.C. & SMITH, J.L. (1983). The ultrastructural localization of immunoglobulin in chronic lymphocytic lymphoma cells: changes in light and heavy chain distribution induced by mitogen stimulation. *Blood*, **61**, 511.

OKAMURA, J., GELFAND, E.W. & LETARTE, M. (1982). Heterogeneity of the response of chronic lymphocytic leukemia cells to phorbol ester. *Blood*, **60**, 1082.

PAPADIMITRIOU, C.S., MULLER-HERMELINK, U. & LENNERT, K. (1979). Histologic and immunohistochemical findings in the differential diagnosis of chronic lymphocytic leukemia of B-cell type and lymphoplasmacytic/lymphoplasmacytoid lymphoma. *Virchows Arch. A. Path. Anat. Histol.*, **384**, 149.

PETO, R., PIKE, M.C., ARMITAGE, P. & 7 others. (1977). Design and analysis of randomised clinical trials requiring prolonged observation of each patient. II. Analysis and examples. *Br. J. Cancer*, **35**, 1.

PLATSOUCAS, C.D., GALINSKI, M., KEMPIN, S., REICH, L., CLARKSON, B. & GOOD, R.A. (1982). Abnormal T lymphocyte subpopulations in patients with B cell chronic lymphocytic leukemia: an analysis by monoclonal antibodies. *J. Immunol.*, **129**, 2305.

PREUD'HOMME, J.L. & SELIGMANN, M. (1972). Surface bound immunoglobulin as a cell marker in human lymphoproliferative diseases. *Blood*, 1972, **40**, 777.

RITZ, J., NADLER, L.M., BHAN, A.K., NOTIS-MCCONARTY, J., PESANDO, J.M. & SCHLOSSMAN, S.F. (1981). Expression of common acute lymphoblastic leukemia antigen (CALLA) by lymphomas of B-cell and T-cell lineage. *Blood*, **58**, 648.

ROSE, M.L., HABESHAW, J.A., KENNEDY, R., SLOANE, J., WILTSHAW, E. & DAVIES A.J.S. (1981). Binding of peanut lectin to germinal centre cells: a marker for B-cell subsets of follicular lymphoma? *Br. J. Cancer*, **44**, 68.

SEMENZATO, G., PEZZUTTO, A., AGOSTINI, C., ALBERTIN, M. & GASPAROTTO, G. (1981). T-lymphocyte subpopulations in chronic lymphocytic leukemia: a quantitative and functional study. *Cancer*, **48**, 2191.

STASHENKO, P., NADLER, L.M., HARDY, R. & SCHLOSSMAN, S.F. (1980). Characterisation of a human B lymphocyte-specific antigen. *J. Immunol.*, **125**, 1678.

STEIN, H., BONK, A., TOLKSDORF, G., LENNERT, K., RODT, H. & GERDES, J. (1980). Immunohistologic analysis of the organization of normal lymphoid tissue and non-Hodgkin's lymphomas. *J. Histochem. Cytochem.*, **28**, 746.

STEIN, H., STAUDINGER, M., TOLKSDORF, G. & LENNERT, K. (1981). Immunologic markers in the differential diagnosis of non-Hodgkin's lymphomas. *J. Cancer Res. Clin. Oncol.*, **101**, 29.

STEIN, H., GERDES, J. & MASON, D.Y. (1982). The normal and malignant germinal centre. *Clin. Haematol.*, **11**, 531.

STEVENSON, F.K., HAMBLIN, T.J. & STEVENSON, G.T. (1981). The nature of the immunoglobulin G on the surface of B lymphocytes in chronic lymphocytic leukemia. *J. Exp. Med.*, **154**, 1965.

STEVENSON, F.K., HAMBLIN, T.J., STEVENSON, G.T. & TUTT, A.L. (1980). Extracellular idiotypic immunoglobulin arising from human leukemic B lymphocytes. *J. Exp. Med.*, **152**, 1484.

STEVENSON, F.K., STEVENSON, G.T. & TUTT, A.L. (1982). The export of immunoglobulin D by human neoplastic B lymphocytes. *J. Exp. Med.*, **156**, 337.

SWERDLOW, S.H., HABESHAW, J.A., MURRAY, L.J., DHALIWAL, H.S., LISTER, T.A. & STANSFELD, A.G. (1983). Centrocytic lymphoma: a distinct clinicopathologic and immunologic entity. A multi-parameter study of 18 cases at diagnosis and relapse. *Am. J. Pathol.*, **113**, 181.

SWERDLOW, S.H., & MURRAY, L.J. (1984). Natural killer (Leu7+) cells in reactive lymphoid tissues and malignant lymphomas. *Am. J. Clin. Pathol.*, **81**, 459.

TOTTERMAN, T.H., NILSSON, K., CLAESSON, L., SIMONSSON, B. & AMAN, P. (1981*a*). Differentiation of chronic lymphocytic leukaemia cells *in vitro*. I. Phorbol ester-induced changes in the synthesis of immunoglobulin and HLA-DR. *Hum. Lymphocyte Diff.*, **1**, 13.

TOTTERMAN, T.H., NILSSON, K. & SUNDSTROM, C. (1980). Phorbol ester-induced differentiation of chronic lymphocytic leukaemia cells. *Nature*, **288**, 176.

TOTTERMAN, T.H., NILSSON, K., SUNDSTROM, C. & SALLSTROM, J. (1981*b*). Differentiation of chronic lymphocytic leukaemia cells *in vitro*. II. Phorbol ester-induced changes in surface marker profile and ultrastructure. *Hum. Lymphocyte Diff.*, **1**, 83.

TUBBS, R.R., FISHLEDER, A., WEISS, R.A., SAVAGE, R.A., SEBEK, B.A. & WEICK, J.K. (1983). Immunohistologic cellular phenotypes of lymphoproliferative disorders. *Am. J. Pathol.*, **11**, 207.

YASUDA, N., KANOH, T., SHIRAKAWA, S. & UCHINO, H. (1982). Intracellular immunoglobulin in lymphocytes from patients with chronic lymphocytic leukemia: an immunoelectron microscopic study. *Leukemia Res.*, **6**, 659.

YUNIS, J.J., OKEN, M.M., KAPLAN, M.E., ENSRUD, K.M., HOWE, R.R. & THEOLOGIDES, A. (1982). Distinctive chromosomal abnormalities in histologic subtypes of non-Hodgkin's lymphoma. *N. Engl. J. Med.*, **307**, 1231.